# Enhancing the Understanding of Abdominal Trauma During the COVID-19 Pandemic Through Co-Occurrence Analysis and Machine Learning

**DOI:** 10.3390/diagnostics14212444

**Published:** 2024-10-31

**Authors:** Dumitru Radulescu, Dan Marian Calafeteanu, Patricia-Mihaela Radulescu, Gheorghe-Jean Boldea, Razvan Mercut, Eleonora Daniela Ciupeanu-Calugaru, Eugen-Florin Georgescu, Ana Maria Boldea, Ion Georgescu, Elena-Irina Caluianu, Georgiana-Andreea Marinescu, Emil-Tiberius Trasca

**Affiliations:** 1 Department of Surgery, University of Medicine and Pharmacy of Craiova, 200349 Craiova, Romania; dr_radulescu_dumitru@yahoo.com (D.R.); ion_georgescu@yahoo.com (I.G.); rui_costa23@yahoo.com (E.-I.C.); etrasca@yahoo.com (E.-T.T.); 2 Department of Ortopedics, The Military Emergency Clinical Hospital ‘Dr. Stefan Odobleja’ Craiova, 200749 Craiova, Romania; 3 Department of Pulmology, University of Medicine and Pharmacy of Craiova, 200349 Craiova, Romania; 4 Doctoral School, University of Medicine and Pharmacy of Craiova, 200349 Craiova, Romania; calugaruanamaria11@yahoo.ro (A.M.B.); marinescu_georgiana.andreea@yahoo.com (G.-A.M.); 5 Department of Plastic and Reconstructive Surgery, University of Medicine and Pharmacy of Craiova, 200349 Craiova, Romania; mercut.razvan@umfcv.ro; 6 Department of Biology and Environmental Engineering, University of Craiova, 200585 Craiova, Romania; ciupeanudaniela@gmail.com

**Keywords:** abdominal trauma, COVID-19 pandemic, co-occurrence, machine learning

## Abstract

Background: This study examines the impact of the COVID-19 pandemic on abdominal trauma management by comparing pre-pandemic (17 February 2018–26 February 2020) and pandemic periods (27 February 2020–7 March 2022). Methods: Analyzing data from 118 patients at the Emergency County Clinical Hospital of Craiova, we identified significant shifts in clinical practices affecting patient outcomes. Results: During the pandemic, a moderate increase in surgical interventions for specific abdominal traumas indicated the effective adaptation of the medical system. Prioritizing critical cases and deferring non-urgent procedures optimized limited resources. Demographic and clinical factors—including age, sex, body mass index (BMI), and red cell distribution width (RDW)—significantly influenced the hospitalization duration and recovery outcomes. Gender disparities in mortality lessened during the pandemic, possibly due to standardized interventions and the physiological effects of SARS-CoV-2. The link between occupation and obesity highlighted how work environments impact trauma severity, especially as lifestyle changes affect BMI. While age remained a major predictor of mortality, its influence slightly decreased, potentially due to improved protocols for elderly patients. RDW emerged as an important prognostic marker for disease severity and mortality risk. Conclusions: Employing advanced co-occurrence analysis enhanced with machine learning, we uncovered complex relationships between clinical and demographic variables often overlooked by traditional methods. This innovative approach provided deeper insights into the collective impact of various factors on patient outcomes. Our findings demonstrate the healthcare system’s rapid adaptations during the pandemic and offer critical insights for optimizing medical strategies and developing personalized interventions in global crises.

## 1. Introduction

The first cases of COVID-19 were reported in December 2019 and were associated with a seafood market in Wuhan, China, where live wild animals were also sold. This connection suggested the potential transmission of the virus from wild animals to humans [1]. Due to the rapid global spread of the virus and its profound impact on communities and countries worldwide, the World Health Organization (WHO) officially declared COVID-19 a pandemic on 11 March 2020 [2]. Studies have demonstrated that the pandemic negatively affected waiting times for medical and surgical interventions, as well as access to essential treatments [3,4,5,6]. The emergence of the COVID-19 pandemic temporarily overshadowed another ongoing global health crisis: the trauma pandemic [7].

An injury is defined as damage to the body’s tissues, whether intentional or accidental, resulting from sudden exposure to various types of energy (mechanical, thermal, electrical, chemical, and radiation) that exceed the physiological tolerance threshold, leading to cell death or homeostatic imbalances [8]. Traumatic injuries are the leading cause of death among individuals aged 1 to 44, with a significant impact on children and young adults due to their active lifestyles and differing mechanisms of injury compared to older age groups [9,10]. The proportion of elderly individuals in the global population is increasing due to higher life expectancy and declining birth rates, leading to an aging population in many countries and, consequently, altering future trauma cohorts [11,12,13].

Abdominal trauma is rare, occurring in less than 10% of all trauma patients, with the liver, spleen, and kidneys being the most frequently affected organs. Given the vital organs involved in the abdominal region, the overall mortality rate can be as high as 20%. Variations in the morbidity and mortality rates are influenced by a range of factors, from patient characteristics and epidemiological context to medical facilities and surgical expertise [14,15].

During the COVID-19 pandemic, the management of trauma patients in healthcare facilities faced significant challenges related to infrastructure, medical personnel, and patient care, all while attempting to prevent the spread of the virus. Additionally, the presence of COVID-19 complicated the interpretation of signs, symptoms, and tests, as the effects of the virus could mask or confuse the diagnosis and appropriate treatment of traumatic injuries [16,17].

Following a traumatic event, the body initiates a cascade of physiological responses that disrupt the homeostasis of the immune system and increase susceptibility to opportunistic infections, a process that can culminate in death if not effectively managed. Similar inflammatory processes have been observed in COVID-19 infections. However, in the context of trauma, COVID-19 infection acts as a catalyst, amplifying the inflammatory response and its consequences [18]. The resulting inflammation is a protective biological response of the immune system to harmful exogenous and endogenous stimuli, aimed at eliminating the initial cause of cellular injury, removing damaged cells and tissues, and establishing the necessary conditions for tissue repair. However, prolonged or excessive inflammatory responses can lead to tissue damage and are a significant factor in the development of many chronic diseases [19,20].

The aim of this study is to evaluate and compare the manifestations of complications and mortality among patients with abdominal trauma before and during the pandemic, exploring the interaction between injury severity and discharge outcomes. By analyzing these changes, we aim to gain a better understanding of the impact of the COVID-19 pandemic on the management of abdominal trauma. This endeavor may facilitate the optimization of medical interventions and contribute to improved patient outcomes by providing essential data for clinical approaches adapted to pandemic conditions.

Furthermore, the study introduces an innovative methodology that integrates co-occurrence matrices with advanced machine learning techniques. This approach allows the identification of complex relationships and hidden patterns that might remain undetected through traditional statistical methods. By applying these techniques, our study not only enhances the precision of clinical analyses but also opens new avenues for optimizing patient treatment and management, particularly during crises such as the COVID-19 pandemic.

## 2. Materials and Methods

### 2.1. Study Design

This retrospective study evaluated consecutive cases of abdominal trauma (AT) treated in three university-affiliated general surgery departments at the Clinical Emergency County Hospital of Craiova, following prior approval from the Ethics Committee of each unit. Patient data were collected from the hospital’s trauma registry using the diagnostic codes S36.X, S37.X, S38.X, and S39.X, and only cases with sufficient information for complete validation were included in the study.

After applying the inclusion criteria, data from 118 patients over the age of 16, who sustained abdominal trauma and were admitted to the surgery department, were analyzed. These patients were divided into two distinct groups for analysis based on a significant temporal criterion. The pre-COVID group includes patients admitted between 17 February 2018 and 26 February 2020. The COVID group comprises patients admitted between 27 February 2020, the date marking the first day of the COVID-19 pandemic in Romania, and 7 March 2022, the last day of the pandemic according to official statements from Romanian authorities.

**Figure 1:**
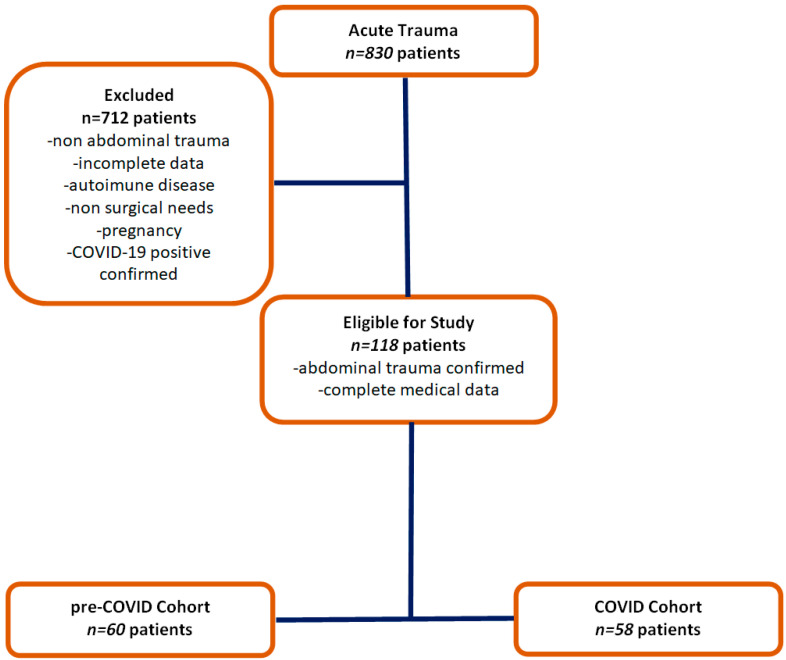
Flow chart of patient inclusion.

This temporal division was established to enable a relevant comparative analysis between the pre-pandemic and pandemic periods, considering the major impact of the pandemic on the healthcare system and, consequently, on the management of abdominal trauma cases. Patients with trauma located in anatomical regions other than the abdomen and those with incomplete data were excluded from the analysis (Figure 1).

Comprehensive demographic data, including sex, age, insurance status, body mass index (BMI), and relevant comorbidities, such as diabetes, congestive heart failure, stroke, myocardial infarction, coronary artery disease, cancer, chronic renal failure, chronic obstructive pulmonary disease, dementia, cirrhosis, and smoking habits, were collected.

At the time of admission, injury characteristics were documented, analyzing trauma mechanisms such as falls from height, pedestrian injuries, motorcycle collisions, motor vehicle accidents, assaults, sports injuries, suicide attempts, and domestic violence. Injury severity was assessed using the Injury Severity Score (ISS) and the Abbreviated Injury Scale (AIS) for various anatomical regions.

Upon presentation, vital signs and other physical examination results were recorded, including systolic blood pressure, respiratory rate, pulse, temperature, and Glasgow Coma Scale score.

The outcomes included the length of hospital stay, the duration of intensive care unit (ICU) admission, days of mechanical ventilation, the volume of packed red blood cells and fresh frozen plasma transfused within the first four hours, and the surgical interventions performed. Complications such as sepsis, stroke, myocardial infarction, pneumonia, acute renal failure, deep vein thrombosis, pulmonary embolism, delirium tremens, and mortality were also monitored.

Complete blood counts (CBCs) included white blood cell, neutrophil, lymphocyte, monocyte, and red blood cell counts, hemoglobin levels, hematocrit, mean corpuscular volume, red cell distribution width, platelet count, mean platelet volume, and routine biochemical analyses. Additionally, inflammatory biomarkers and COVID-19 infection status were analyzed. This information was used to evaluate and compare clinical outcomes and to examine the evolution of abdominal trauma patients in the context of the COVID-19 pandemic.

### 2.2. Inclusion Criteria

To be included in the study, the patients were required to have confirmed abdominal trauma by one or more of the following methods: detailed clinical evaluation, diagnostic imaging (CT, MRI, or ultrasound), or surgical interventions confirming the presence of trauma. Only the patients with complete sets of clinical and paraclinical data, essential for the full validation of each case according to the study’s objectives, were selected.

### 2.3. Exclusion Criteria

The patients who sustained traumatic injuries outside the abdominal region or whose injuries did not directly affect the abdominal organs were excluded from the study. Additionally, patients with incomplete medical documentation at the time of admission or with missing data necessary for evaluation during treatment were not included.

To avoid confounding effects related to complications specific to COVID-19 infection, the patients diagnosed with COVID-19 were excluded from the analysis, ensuring the consistency and accuracy of the results. Patients with autoimmune diseases were also excluded due to the increased risk of complications and potential negative interactions with abdominal trauma. Pregnant women were not included in the study due to the specific treatment needs and risks associated with pregnancy, as well as the potential impact of abdominal trauma on pregnancy outcomes.

### 2.4. Statistical Analysis

Statistical analysis was performed using standard methods to evaluate the distribution of data. The Shapiro–Wilk test was applied to assess the normality of the distribution, thereby ensuring the appropriateness of the subsequent statistical methods. Upon the confirmation of normality, chi-square and Mann–Whitney tests were employed to identify significant differences between the groups, with a significance threshold set at *p* < 0.05.

Data analysis was conducted using SPSS version 26.0 (IBM Corporation, Armonk, NY, USA), chosen for its advanced capabilities and widespread use in medical research. The data were expressed as the median and interquartile range (IQR) to provide a more accurate representation of the distribution.

In addition, to explore the complex relationships between variables, machine learning techniques were implemented using Python version 3.9. A feed-forward neural network model was used to generate data representations and calculate the co-occurrence matrix. This approach facilitated the identification of complex patterns and interdependencies among the variables studied.

To ensure the reproducibility of the results, a random seed was set to 42, a common practice in scientific research, allowing for consistent outcomes with each execution of the code. While the exact values of correlations may vary slightly between runs, significant and robust correlations persist, suggesting that the identified models reflect genuine interactions among the variables studied.

By leveraging machine learning, we overcame the limitations of traditional statistical analysis, successfully uncovering complex patterns and interdependent relationships among the analyzed variables, thereby enhancing the understanding of interaction dynamics in the context of abdominal trauma during the COVID-19 pandemic.

All the analyses were performed using Python 3.9 and the libraries TensorFlow 2.13.0, NumPy 1.24.4, and SciPy 1.10.1.

In the context of using machine learning techniques to construct the co-occurrence matrix, the diagonal values are not necessarily equal to 1, as these values reflect the learned relationships between individual variables within a complex dataset, rather than merely their simultaneous presence. Unlike traditional approaches, where the diagonal might represent a perfect self-coincidence (resulting in values of 1), machine learning integrates multiple influences and interdependencies between variables, which can alter these values. This allows the model to capture not only the presence or absence of a variable but also the degree and nature of its relationship with other variables, providing a more nuanced view of the interactions in the analyzed data.

### 2.5. Machine Learning Model Validation

To ensure the performance and generalization capability of the machine learning model used in generating the co-occurrence matrix, a rigorous validation process was implemented.

The model used was a feed-forward neural network (FFNN), constructed and trained with the TensorFlow/Keras library. The network consists of an input layer; two fully connected hidden layers with 64 and 32 units, respectively, each followed by ReLU (Rectified Linear Unit) activation functions; and a final softmax layer to compute the probability distribution across the target classes.

The model was trained using the Adam optimization algorithm, with a learning rate of 0.001, over 10 epochs, and a batch size of 32. To reduce the risk of overfitting, we implemented dropout layers with a dropout rate of 0.5 after each hidden layer. Additionally, early stopping was applied, which monitors validation loss during training and halts the process if there is no improvement, ensuring the model does not overfit to the training data.

The dataset was split into two subsets: 80% for training and 20% for testing. This split allowed the evaluation of the model on unseen data, providing a realistic estimate of the model’s ability to generalize the relationships between variables. To reduce the risk of overfitting and assess model stability, we applied 5-fold cross-validation. In this method, the data were divided into five subsets, and the model was trained on four of these and tested on the fifth, repeating the process for each fold. This allowed every instance in the dataset to be used for both training and testing, ensuring a comprehensive evaluation of the model’s performance.

To prevent overfitting to the training data, we employed techniques such as dropout and early stopping, which helped maintain a balance between the model’s learning and its generalization ability. The model’s performance was measured using relevant metrics such as the following :

Accuracy: the overall percentage of correct predictions;

Precision: the percentage of correct positive predictions out of all the positive predictions;

Recall: the proportion of actual positive instances correctly identified;

F1 Score: the harmonic mean between precision and recall, particularly useful in cases of class imbalance.

To analyze the impact of demographic, clinical, and laboratory markers on clinical outcomes across the pre- and post-pandemic periods, we employed the LIME (Local Interpretable Model-Agnostic Explanations) technique. LIME allows for a detailed assessment of how individual features contribute to model predictions by generating locally interpretable explanations. This method was instrumental in identifying nuanced changes between the two periods, offering deeper insights into variable interactions that traditional models, such as logistic regression, might not fully capture.

## 3. Results

A retrospective cohort of 830 patients who presented to the emergency department and were diagnosed with various traumas between 1 January 2016 and 3 December 2021 was included in the study. Of these, 118 patients were selected based on strict inclusion criteria for detailed analysis and were divided into pre-COVID and COVID groups, as described in the study design. This study investigates the impact of the COVID-19 pandemic on trauma patients, assessing differences between the pre-pandemic and pandemic periods (Table 1). This division aimed to identify and compare clinical trends and outcomes within the context of the COVID-19 pandemic.

In the univariate analysis, among all the included clinical parameters, only the variable hemoperitoneum was statistically significant, with an Odds Ratio of 2.66 (95% CI: 1.26, 5.61) and a *p*-value of 0.0102. In the multivariate analysis, we considered variables such as obesity, hypertension, surgery, and sex, which, although they did not reach statistical significance, had *p*-values close to significance and are relevant in our clinical context.

However, the apparent similarity of data between the two periods limited the ability of logistic regression to capture subtle differences. Therefore, we decided not to repeat the logistic regression analysis for biological parameters to avoid overburdening the article with redundant analyses. Instead, we turned to advanced co-occurrence and machine learning methods to highlight the complex relationships between the variables and capture more nuanced differences between the periods.

### 3.1. Analysis of Demographic Factors and Clinical Outcomes in the Context of Pre- and Post-Pandemic Abdominal Trauma

Our study investigated the influence of demographic factors on the clinical outcomes of patients with abdominal trauma across two distinct periods: pre-pandemic and pandemic. The analysis incorporated the use of co-occurrence matrices and classical statistical methods to evaluate the relationships between demographic variables and clinical outcomes.

**Figure 2:**
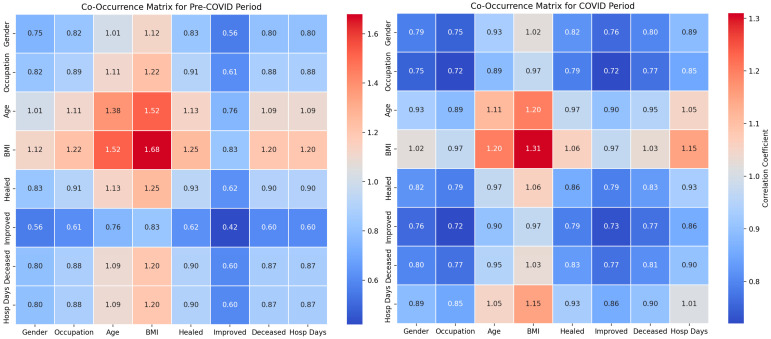
Demographic impact co-occurrence matrix in Abdominal Traumatology.

The classical statistical analysis did not identify significant differences related to sex between the pre-COVID and COVID periods (*p* = 0.46) (Table 2), suggesting that sex did not significantly influence the clinical outcomes during the pandemic. The co-occurrence between sex and recovery was 0.79 pre-COVID and 0.76 during the COVID period, while the co-occurrence between sex and mortality was 0.84 pre-COVID and 0.82 during the COVID period (Figure 2). These results indicate a moderate influence with no significant changes between the two periods.

Occupation did not show significant differences between the pre-COVID and COVID periods (*p* = 0.74), indicating stability in occupational risk factors. The co-occurrence matrices revealed a strong co-occurrence between occupation and BMI (1.21 pre-COVID and 1.06 during the COVID period), suggesting a significant correlation. The co-occurrence between occupation and mortality was 0.95 pre-COVID and 0.85 during the COVID period, implying that occupation may affect the risk and severity of trauma.

The classical statistical analysis indicated a non-significant trend toward an increased impact of age during the pandemic (*p* = 0.11). The co-occurrence between age and BMI was 1.43 pre-COVID and 1.29 during the COVID period, while the co-occurrence between age and mortality was 1.11 pre-COVID and 1.03 during the COVID period, suggesting a significant influence of age on the mortality and hospitalization duration.

The classical statistical analysis revealed that the prevalence of obesity increased significantly during the pandemic (*p* = 0.09). The co-occurrence between BMI and days of hospitalization was 1.27 pre-COVID and 1.11 during the COVID period. Additionally, the co-occurrence between BMI and mortality was 1.30 pre-COVID and 1.16 during the COVID period. These data suggest that obesity significantly influences mortality and the duration of treatment.

The classical statistical analysis showed a decrease in the co-occurrence of days of hospitalization and mortality during the COVID period, suggesting an adaptation of clinical protocols and increased efficiency in managing emergencies. In the pre-COVID period, the co-occurrence between BMI and days of hospitalization was 1.27, which decreased to 1.11 during the COVID period. Furthermore, the waiting time for treatment decreased during the pandemic, with a co-occurrence of 0.27 pre-COVID and 0.15 during the COVID period, indicating an improvement in the initial management of patients.

The classical statistical analysis revealed a reduction in waiting time for treatment and an increase in the efficiency of the emergency system, reflecting the necessary adaptations in the context of the pandemic. In the pre-COVID period, the median waiting time was 3.0 h (IQR: 1.1–12.2), while during the COVID period, the median was 2.4 h (IQR: 1.3–6.4). These observations underscore the changes in the clinical protocols and resource allocation strategies during the pandemic and their impact on clinical outcomes.

**Figure 3:**
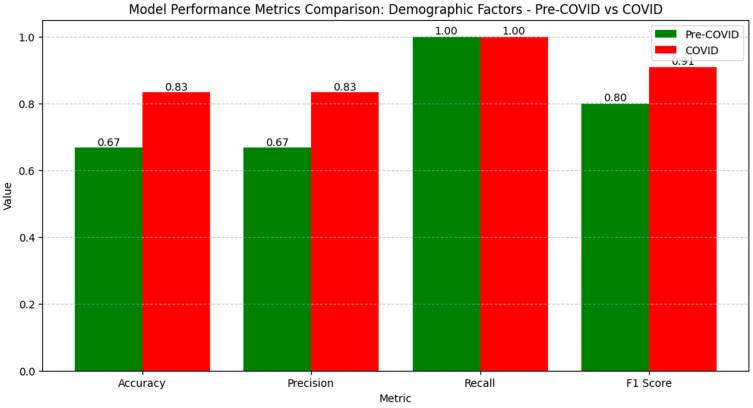
Model performance metrics on the demographic impact of abdominal trauma before and during the pandemic.

The metric results for generating matrices with demographic factors during the pre-COVID and COVID periods show a significant improvement in model performance during the pandemic, indicating an effective adaptation to new conditions (Figure 3).

Accuracy increased from 0.67 to 0.83, and precision rose from 0.67 to 0.83, suggesting the better identification of correct cases and a reduction in false positive results. Recall remained constant at 1.00 in both periods, demonstrating a stable capacity to capture all the relevant cases. The F1 Score increased from 0.80 to 0.91, reflecting a better balance between precision and recall, indicating optimized model performance in analyzing demographic factors in the pandemic context.

**Figure 4:**
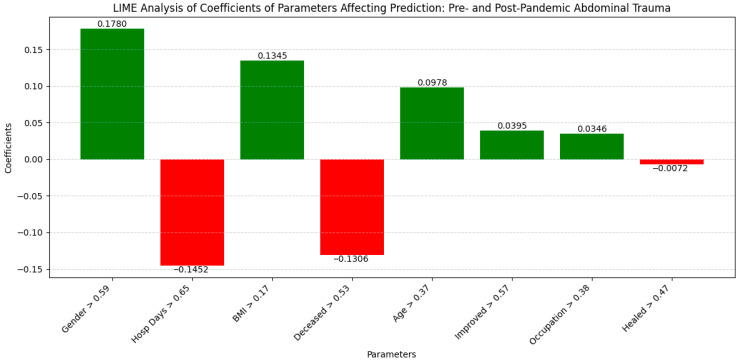
LIME analysis of coefficients of parameters affecting prediction: pre- and post-pandemic demographic impact.

The LIME analysis identified several key correlations influencing the clinical outcomes, which largely align with the co-occurrence matrix findings despite minor variations (Figure 4).

Gender (0.1780; >0.59) and BMI (0.1345; >0.17) showed moderate positive impacts, highlighting their significant roles. Hospital Days (−0.1452; >0.65) and Deceased (−0.1306; >0.53) exhibited notable negative correlations, reflecting increased treatment efficiency and reduced mortality during the pandemic. Age (0.0978; >0.37) and Improved (0.0395; >0.57) had slight positive influences, while Occupation (0.0346; >0.38) showed a minimal positive effect. Healed (−0.0072; >0.47) had an insignificant impact. These importance thresholds emphasize the relevant contributions of each factor, confirming the robustness of the findings and the consistent influence of the demographic variables on the clinical outcomes before and after the pandemic.

### 3.2. Impact of Trauma Types on Clinical Outcomes Pre- and Post-Pandemic

**Figure 5:**
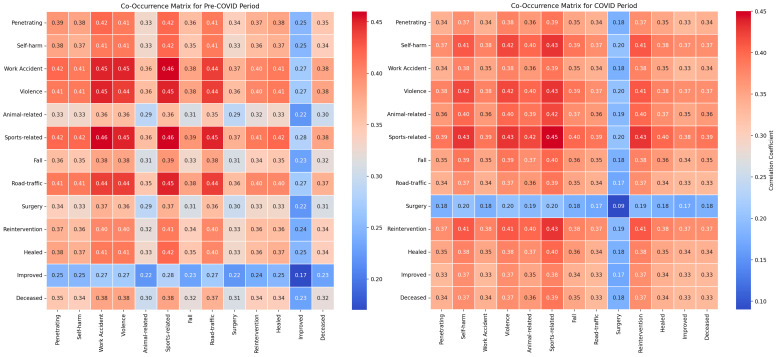
Pre- and post-COVID Abdominal Traumatology management strategy comparison co-occurrence matrix.

Our study investigated the impact of various trauma types on the clinical outcomes of patients with abdominal trauma during the pre-pandemic and pandemic periods utilizing co-occurrence matrices and classical statistical analysis methods. For penetrating traumas, the classical statistical analysis did not indicate significant differences between the periods (*p* = 0.923), with co-occurrence with mortality remaining stable (0.40 pre-pandemic and 0.39 pandemic), and co-occurrence with surgical reinterventions being consistent (0.41 in both periods) (Figure 5).

Regarding self-inflicted injuries, no significant differences were observed between the periods according to the classical statistical analysis (*p* = 0.948). The co-occurrence with mortality was 0.39 pre-pandemic and 0.38 pandemic, while the co-occurrence with surgical reinterventions remained at 0.40 in both periods.

In the analysis of work-related injuries, no significant differences were highlighted between the periods (*p* = 0.980), with co-occurrence with mortality being 0.40 pre-pandemic and 0.39 pandemic, and co-occurrence with surgical reinterventions remaining at 0.41 in both periods.

For violent traumas, the classical statistical analysis did not show significant variations between the periods (*p* = 0.578), with the co-occurrence of mortality remaining constant at 0.38 in both periods, and the co-occurrence with surgical reinterventions at 0.39.

In the case of animal-related injuries, no statistically significant differences were identified between the periods (*p* = 0.751), with co-occurrence with mortality being 0.43 pre-pandemic and 0.42 pandemic, while co-occurrence with surgical reinterventions varied slightly (0.44 pre-pandemic and 0.43 pandemic).

The reduced number of sports-related cases during the pandemic limited the ability to evaluate the statistical significance of sports traumas. The co-occurrence with mortality was 0.38 in both periods, and the co-occurrence with surgical reinterventions remained stable at 0.39.

For falls, the classical statistical analysis did not reveal significant differences between the periods (*p* = 0.767), with co-occurrence with mortality being 0.38 pre-pandemic and 0.37 pandemic, and co-occurrence with surgical reinterventions remaining constant at 0.39.

Finally, the analysis of road traffic traumas did not show significant differences between the periods (*p* = 0.900), with co-occurrence with mortality being 0.40 pre-pandemic and 0.38 pandemic, and co-occurrence with surgical reinterventions remaining constant at 0.39 in both periods.

**Figure 6:**
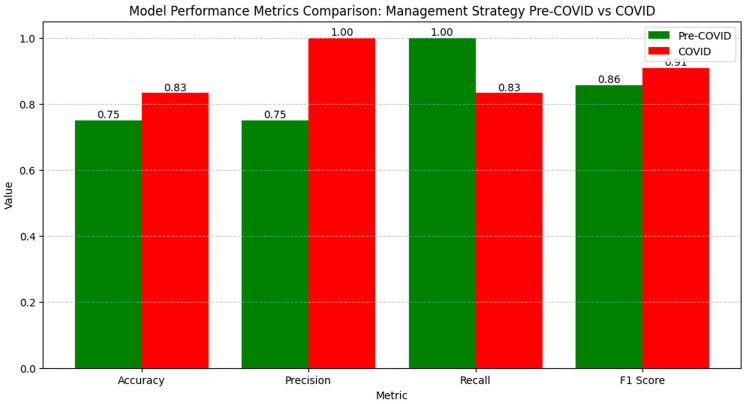
Model performance metrics on the management strategy of abdominal trauma before and during the pandemic.

The numerical values of the metrics for evaluating clinical outcomes indicate significant variations between the pre-COVID and COVID periods (Figure 6).

Accuracy increased from 0.75 to 0.83, reflecting an overall improvement in correctly classifying cases during the pandemic. Precision rose from 0.75 to 1.00, showing a significant reduction in false positive results. However, recall decreased from 1.00 to 0.83, suggesting a slight decline in capturing all the relevant cases. The F1 Score increased from 0.86 to 0.91, indicating an improved balance between precision and recall, suggesting that the model managed to optimize performance in evaluating clinical outcomes in the pandemic context.

**Figure 7:**
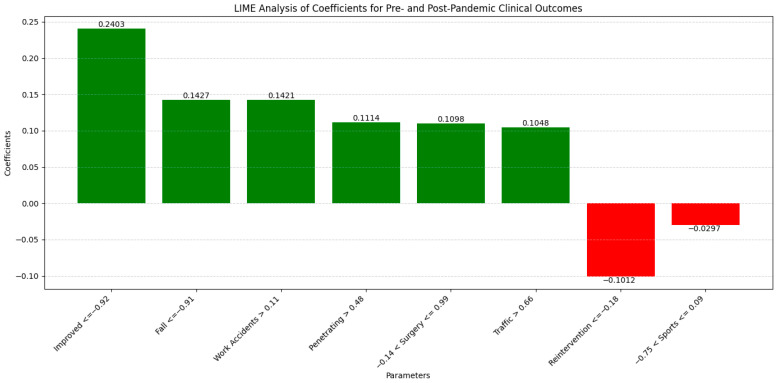
LIME analysis of coefficients of parameters affecting prediction: pre- and post-pandemic management strategy.

The LIME analysis results largely mirror those from the co-occurrence matrix, indicating the stable influences of trauma types on clinical outcomes before and after the pandemic, with no significant differences between the periods. To streamline our analysis, we present the LIME values for the eight leading parameters (Figure 7).

Improved (0.2403; ≤−0.92) showed a moderate positive influence, aligning with the matrix which found no significant period differences. Fall (0.1427; ≤−0.91) exhibited a consistent positive impact, reflecting stable co-occurrence with mortality and surgical interventions across both periods. Work Accidents (0.1421; >0.11) demonstrated a moderate positive correlation, corroborating the matrix’s findings of unchanged associations with mortality and surgeries. Penetrating Trauma (0.1114; >0.48) maintained a moderate positive influence, consistent with stable co-occurrence metrics. Operation (0.1098; −0.14 ≤ Operation ≤ 0.99) indicated a moderate positive contribution, matching the matrix’s stable surgical intervention correlations. Road Traffic (0.1048; >0.66) showed a slight positive impact, consistent with steady co-occurrence with mortality and surgeries. Reintervention (−0.1012; ≤−0.18) presented a slight negative influence, aligning with the matrix’s stable reintervention rates. Sport-Related (−0.0297; −0.75 < Sport-Related ≤ 0.09) exhibited a minimal negative impact, reflecting the matrix’s stable but limited associations due to fewer cases during the pandemic. Overall, these correlations confirm that the types of trauma have maintained their influence on clinical outcomes consistently across both the pre- and post-pandemic periods, validating the findings from both the co-occurrence matrix and LIME analysis.

### 3.3. Impact of Comorbidities and Surgical Interventions on Clinical Outcomes Pre- and Post-Pandemic

**Figure 8:**
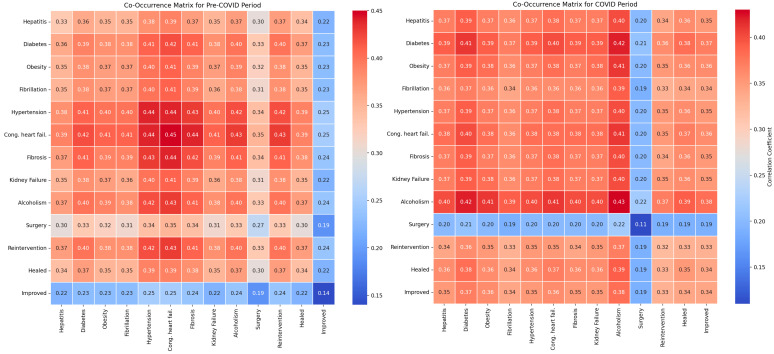
Analysis of the impact of comorbidities on the management of abdominal trauma before and during the pandemic.

Our research evaluated the impact of comorbidities and surgical interventions on the clinical outcomes of patients with abdominal trauma using co-occurrence matrices and classical statistical analysis. For hepatitis, the classical statistical analysis did not reveal significant differences between the periods (*p* = 0.294), with co-occurrence with recovery being 0.41 pre-pandemic and 0.38 pandemic (Figure 8).

For diabetes, the classical statistical analysis did not show relevant differences between the periods (*p* = 0.455), with a co-occurrence of recovery at 0.40 pre-pandemic and 0.38 pandemic.

The classical statistical analysis indicated a significant increase in the prevalence of obesity during the pandemic (*p* = 0.090), with co-occurrence with recovery being 0.38 pre-pandemic and 0.37 pandemic.

Regarding atrial fibrillation, the classical statistical analysis did not reveal significant differences between the periods (*p* = 0.677), with co-occurrence with recovery remaining constant at 0.36 in both periods.

The classical statistical analysis showed a non-significant trend towards an increase in the prevalence of hypertension during the pandemic (*p* = 0.081), with co-occurrence with recovery being 0.41 pre-pandemic and 0.38 pandemic.

For congestive heart failure, the classical statistical analysis did not show significant differences between the periods (*p* = 0.751), with co-occurrence with recovery being 0.39 in both periods.

Regarding fibrosis, the classical statistical analysis did not indicate significant variations between the periods (*p* = 0.706), with co-occurrence with recovery being 0.42 pre-pandemic and 0.40 pandemic.

For renal failure, the classical statistical analysis did not reveal notable differences between the periods (*p* = 0.706), with co-occurrence with recovery remaining constant at 0.41 in both periods.

Regarding alcoholism, the classical statistical analysis did not reveal significant differences between the periods (*p* = 0.699), with co-occurrence with recovery being 0.42 pre-pandemic and 0.40 pandemic.

The classical statistical analysis did not show significant differences between the periods for surgical interventions (*p* = 0.144), with co-occurrence with recovery being 0.38 pre-pandemic and 0.37 pandemic.

For surgical reinterventions, the classical statistical analysis did not indicate relevant differences between the periods (*p* = 0.341), with co-occurrence with recovery remaining stable at 0.40 in both periods.

**Figure 9:**
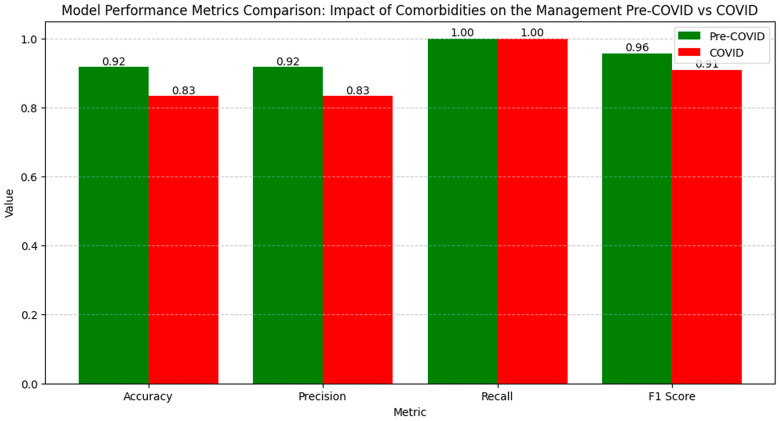
Model performance metrics on the impact of comorbidities on the management of abdominal trauma before and during the pandemic.

The numerical values of the metrics for evaluating the impact of comorbidities and surgical interventions on clinical outcomes show a slight variation in model performance between the pre-pandemic and pandemic periods (Figure 9).

Accuracy slightly decreased from 0.92 pre-COVID to 0.83 during the COVID period, indicating a small reduction in correctly classifying cases. Precision also decreased from 0.92 to 0.83, reflecting an increase in false positive results during the pandemic. Recall remained constant at 1.00 in both periods, showing that the model continued to capture all the relevant cases. The F1 Score decreased from 0.96 pre-COVID to 0.91 during the COVID period, indicating a slight decrease in the balance between precision and recall, suggesting a minor variation in the overall performance of the model in evaluating the clinical outcomes related to comorbidities and surgical interventions in the pandemic context.

**Figure 10:**
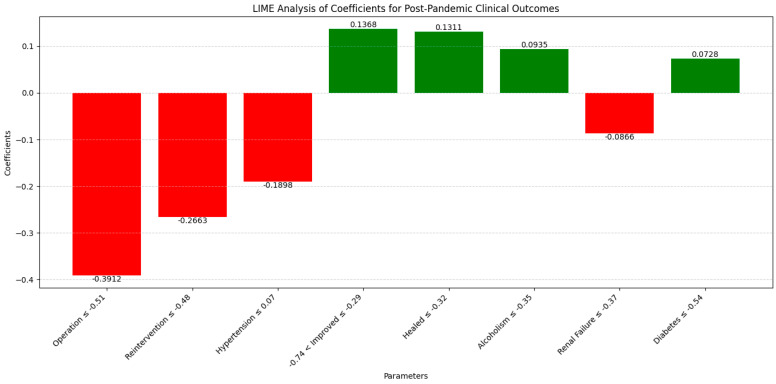
LIME analysis of coefficients of comorbidities affecting prediction: pre- and post-pandemic management strategy.

The LIME analysis results largely mirror those from the co-occurrence matrix, indicating the stable influences of comorbidities and surgical interventions on clinical outcomes post-pandemic, with no significant differences between the periods. We illustrate the eight key parameters derived from the LIME analysis, maintaining a clear and uncluttered presentation in this section (Figure 10).

Operation (−0.3912; ≤−0.51) showed a moderate negative influence on recovery, consistent with the matrix’s similar co-occurrence with recovery (0.38 pre-pandemic and 0.37 pandemic). Reintervention (−0.2663; ≤−0.48) demonstrated a moderate negative impact, aligning with stable co-occurrence (0.40 in both periods). Hypertension (−0.1898; ≤0.07) had a slight negative influence, reflecting the slight decrease in co-occurrence with recovery (0.41 pre-pandemic to 0.38 pandemic). Improved (0.1368; −0.74 < Improved ≤ −0.29) exhibited a moderate positive influence, consistent with constant co-occurrence with recovery, while Healed (0.1311; ≤−0.32) showed a moderate positive effect, reflecting stable recovery outcomes. Alcoholism (0.0935; ≤−0.35) had a slight positive impact, consistent with stable co-occurrence (0.42 pre-pandemic and 0.40 pandemic). Renal Failure (−0.0866; ≤−0.37) indicated a minor negative influence, matching the stable impact on recovery (0.41 in both periods), and Diabetes (0.0728; ≤−0.54) presented a slight positive effect, aligned with stable co-occurrence (0.40 pre-pandemic and 0.38 pandemic). Overall, these correlations confirm that the influences of comorbidities and surgical interventions on clinical outcomes remained consistent between the pre- and post-pandemic periods, validating the findings from both the co-occurrence matrix and LIME analysis.

### 3.4. Analysis of the Influence of Hematological Factors and Comorbidities on Clinical Outcomes in the Pre- and Post-Pandemic Periods

Our study investigated the impact of various hematological parameters and comorbidities on the clinical outcomes of patients with abdominal trauma during the pre-pandemic and pandemic periods. We employed co-occurrence matrices and classical statistical analysis methods to identify significant relationships between these variables (Table 3).

**Figure 11:**
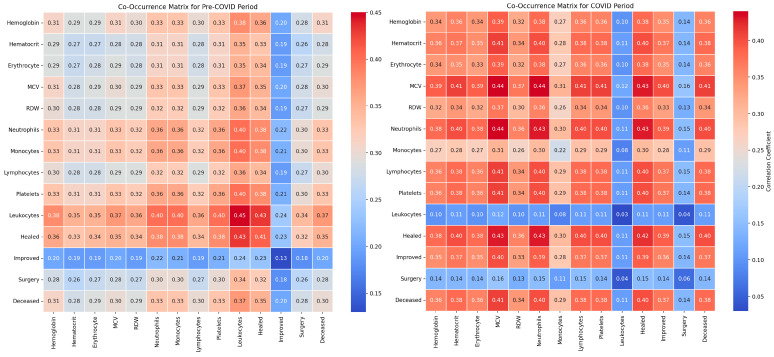
COVID-19 pandemic biologic variable comparative co-occurrence matrix in abdominal trauma response.

The classical statistical analysis did not reveal significant differences between the periods for hemoglobin levels (*p* = 0.948), with co-occurrence with mortality being 0.36 pre-pandemic and 0.42 during the pandemic (Figure 11).

For hematocrit levels, the classical statistical analysis showed no significant variations between the periods (*p* = 0.576), with co-occurrence with mortality being 0.38 pre-pandemic and 0.45 during the pandemic.

The analysis of erythrocyte levels indicated no significant differences between the periods (*p* = 0.421), with co-occurrence with mortality being 0.35 pre-pandemic and 0.38 during the pandemic.

Regarding mean corpuscular volume (MCV) values, the classical statistical analysis did not show significant differences between the periods (*p* = 0.103), with co-occurrence with mortality remaining constant at 0.36 in both periods.

The classical statistical analysis demonstrated a significant increase in the prevalence of RDW during the pandemic (*p* = 0.006), with co-occurrence with mortality being 0.33 pre-pandemic and 0.38 during the pandemic.

For neutrophil count, the classical statistical analysis did not show significant differences between the periods (*p* = 0.739), with co-occurrence with mortality being 0.33 pre-pandemic and 0.39 during the pandemic.

In the case of monocyte count, the classical statistical analysis did not reveal significant differences between the periods (*p* = 0.775), with co-occurrence with mortality being 0.30 pre-pandemic and 0.28 during the pandemic.

The classical statistical analysis did not show significant differences between the periods for lymphocyte count (*p* = 0.557), with co-occurrence with mortality being 0.35 pre-pandemic and 0.42 during the pandemic.

For platelet count, the classical statistical analysis did not indicate significant variations between the periods (*p* = 0.265), with co-occurrence with mortality being 0.34 pre-pandemic and 0.41 during the pandemic.

Regarding leukocyte count, the classical statistical analysis did not indicate significant differences between the periods (*p* = 0.149), with co-occurrence with mortality remaining constant at 0.33 in both periods.

The classical statistical analysis did not reveal significant differences between the periods for surgical interventions (*p* = 0.144), with co-occurrence with recovery being 0.38 pre-pandemic and 0.36 during the pandemic.

For surgical reinterventions, the classical statistical analysis did not indicate significant differences between the periods (*p* = 0.341), with co-occurrence with recovery remaining stable at 0.40 in both periods.

**Figure 12:**
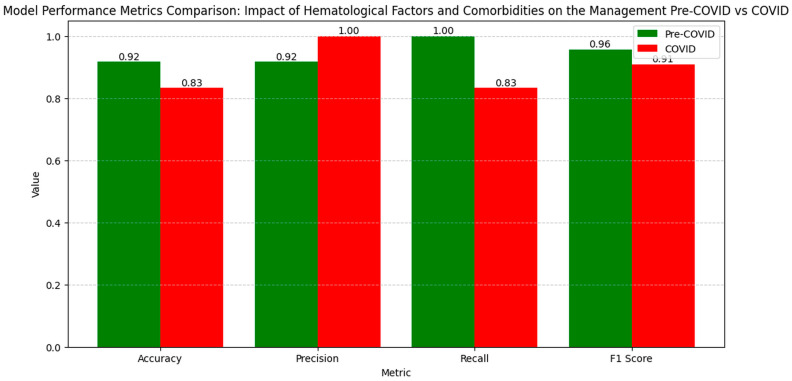
Model performance metrics on the impact of biologic variables on the management of abdominal trauma before and during the pandemic.

The numerical values of the metrics for evaluating the influence of the hematological factors and comorbidities on clinical outcomes show variations in model performance between the pre-pandemic and pandemic periods (Figure 12).

Accuracy decreased from 0.92 pre-COVID to 0.83 during the COVID period, indicating a slight reduction in the model’s ability to correctly classify cases. Precision increased from 0.92 to 1.00, suggesting an improvement in reducing false positive results during the pandemic. However, recall decreased from 1.00 to 0.83, indicating a decline in capturing all the relevant cases. The F1 Score decreased from 0.96 pre-COVID to 0.91 during the COVID period, reflecting a decrease in the balance between precision and recall, suggesting a variation in the overall performance of the model in evaluating the influence of the hematological factors and comorbidities on clinical outcomes in the pandemic context.

**Figure 13:**
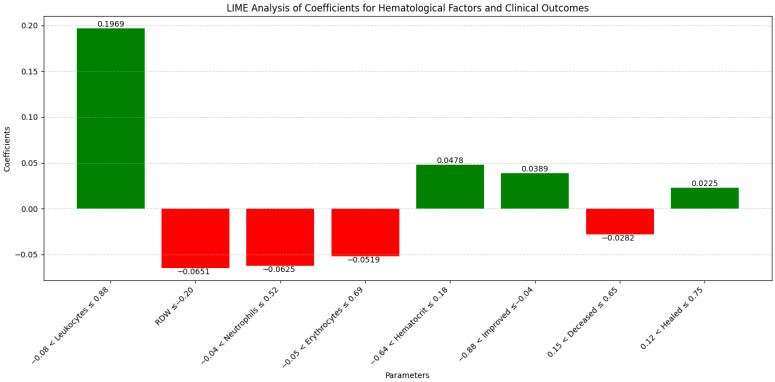
LIME analysis of coefficients of parameters affecting prediction: pre- and post-pandemic biologic variables.

The LIME analysis results largely mirror those from the co-occurrence matrix, indicating a stable influence of the hematological factors and comorbidities on clinical outcomes in both the pre- and post-pandemic periods, with no significant differences between the periods. To maintain clarity and focus, this section highlights the LIME values for the eight most significant parameters (Figure 13).

Specifically, leucocytes (0.1969; −0.08 < Leucocytes ≤ 0.88) showed a moderate positive influence on mortality, consistent with the stable co-occurrence with mortality (0.33 in both periods), confirming that leucocyte count did not significantly change its impact between the periods. RDW (−0.0651; ≤−0.20) exhibited a slight negative influence, aligning with the co-occurrence matrix which showed a moderate increase in RDW co-occurrence with mortality during the pandemic (from 0.33 pre-pandemic to 0.38 pandemic). Neutrophils (−0.0625; −0.04 < Neutrophils ≤ 0.52) demonstrated a slight negative impact, consistent with stable co-occurrence with mortality (0.33 pre-pandemic and 0.39 pandemic), confirming no significant variation in neutrophil impact. Erythrocytes (−0.0519; −0.05 < Erythrocytes ≤ 0.69) indicated a small negative influence, matching the stable co-occurrence with mortality (0.35 pre-pandemic and 0.38 pandemic), highlighting a consistent impact of erythrocyte levels. Hematocrit (0.0478; −0.64 < Hematocrit ≤ 0.18) showed a slight positive influence on mortality, in agreement with the increased co-occurrence during the pandemic (0.38 pre-pandemic to 0.45 pandemic), confirming a slight modification in hematocrit’s impact. Improved (0.0389; −0.88 < Improved ≤ −0.04) presented a positive contribution, consistent with stable co-occurrence across the periods, indicating the consistent influence of improvements on outcomes. Deceased (−0.0282; 0.15 < Deceased ≤ 0.65) revealed a slight negative influence, similar to the co-occurrence matrix which showed a slight increase in mortality co-occurrence during the pandemic for most hematological factors, without significant variations. Healed (0.0225; 0.12 < Healed ≤ 0.75) demonstrated a small positive effect, corresponding with stable co-occurrence for recovery, confirming that the healing process did not significantly differ between the periods. Overall, these correlations emphasize that the influences of the hematological factors and comorbidities on clinical outcomes remained generally stable and consistent between the pre- and post-pandemic periods, validating the conclusions drawn from both the co-occurrence matrix and the LIME analysis.

### 3.5. Correlation of Laboratory Markers with Clinical Outcomes During Pre- and Post-Pandemic Periods

Our study explored the correlation between various laboratory markers and the clinical outcomes of patients with abdominal trauma during the pre-pandemic and pandemic periods. We utilized co-occurrence matrices to identify significant relationships between these markers and clinical variables such as recovery, improvement, the necessity for surgical interventions, and mortality.

**Figure 14:**
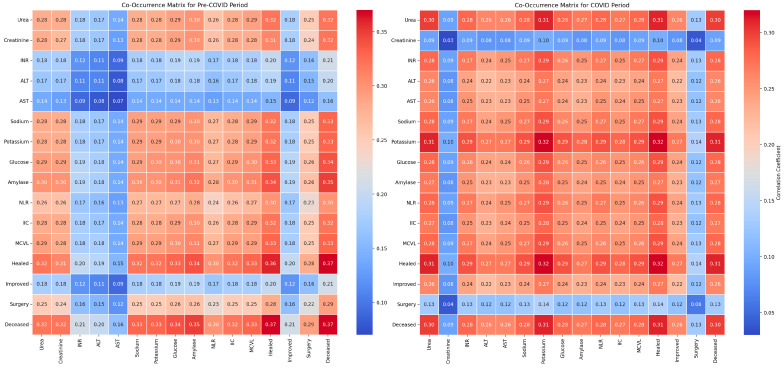
Pre-COVID versus COVID-19 pandemic comparative co-occurrence matrix of biochemical markers in abdominal trauma.

The classical statistical analysis did not reveal significant differences between the periods for urea levels (*p* = 0.850), with co-occurrence with mortality being 0.34 pre-pandemic and 0.30 during the pandemic (Figure 14).

For creatinine levels, the classical statistical analysis showed no significant variations between the periods (*p* = 0.712), with co-occurrence with mortality being 0.32 pre-pandemic and 0.09 during the pandemic.

In the case of International Normalized Ratio (INR) values, the classical statistical analysis indicated a non-significant trend towards an increase during the pandemic (*p* = 0.067), with co-occurrence with mortality being 0.21 pre-pandemic and 0.31 during the pandemic.

For Alanine Aminotransferase (ALT) values, the classical statistical analysis did not show significant differences between the periods (*p* = 0.661), with co-occurrence with mortality being 0.22 pre-pandemic and 0.31 during the pandemic.

The classical statistical analysis did not reveal significant differences between the periods for Aspartate Aminotransferase (AST) values (*p* = 0.486), with co-occurrence with mortality being 0.15 pre-pandemic and 0.28 during the pandemic.

Regarding sodium levels, the classical statistical analysis did not show significant variations between the periods (*p* = 0.545), with co-occurrence with mortality being 0.35 pre-pandemic and 0.31 during the pandemic.

For potassium levels, the classical statistical analysis did not indicate significant differences between the periods (*p* = 0.976), with co-occurrence with mortality being 0.35 pre-pandemic and 0.30 during the pandemic.

The classical statistical analysis did not show significant differences between the periods for glucose levels (*p* = 0.626), with co-occurrence with mortality being 0.32 pre-pandemic and 0.29 during the pandemic.

In the case of amylase levels, the classical statistical analysis did not reveal significant differences between the periods (*p* = 0.353), with co-occurrence with mortality being 0.34 pre-pandemic and 0.30 during the pandemic.

For Neutrophil–Lymphocyte Ratio (NLR) values, the classical statistical analysis did not show significant variations between the periods (*p* = 0.732), with co-occurrence with mortality being 0.33 pre-pandemic and 0.29 during the pandemic.

The classical statistical analysis did not indicate significant differences between the periods for Cumulative Inflammatory Index (IIC) values (*p* = 0.339), with co-occurrence with mortality being 0.32 pre-pandemic and 0.29 during the pandemic.

Regarding MCV–Lymphocyte Ratio (MCVL) values, the classical statistical analysis did not reveal significant differences between the periods (*p* = 0.559), with co-occurrence with mortality being 0.36 pre-pandemic and 0.33 during the pandemic.

**Figure 15:**
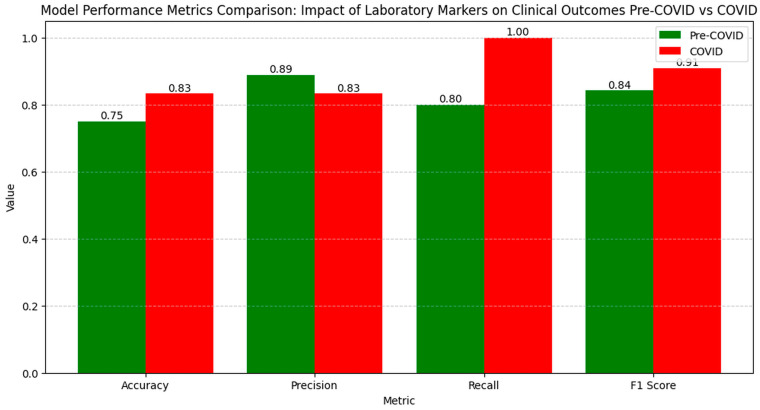
Model performance metrics on the impact of biochemical markers on the management of abdominal trauma before and during the pandemic.

The numerical values of the metrics for evaluating the impact of laboratory markers on clinical outcomes show an improvement in model performance during the COVID period compared to the pre-COVID period (Figure 15).

Accuracy increased from 0.75 pre-COVID to 0.83 during the COVID period, indicating an enhanced ability to correctly classify cases. Precision slightly decreased from 0.89 to 0.83, reflecting a small increase in false positive results. Recall significantly increased from 0.80 pre-COVID to 1.00 during the COVID period, demonstrating a complete capacity to identify all the relevant cases during the pandemic. The F1 Score increased from 0.84 pre-COVID to 0.91 during the COVID period, highlighting an improved balance between precision and recall, suggesting that the model successfully optimized performance in evaluating the influence of laboratory markers on clinical outcomes during the pandemic period.

**Figure 16:**
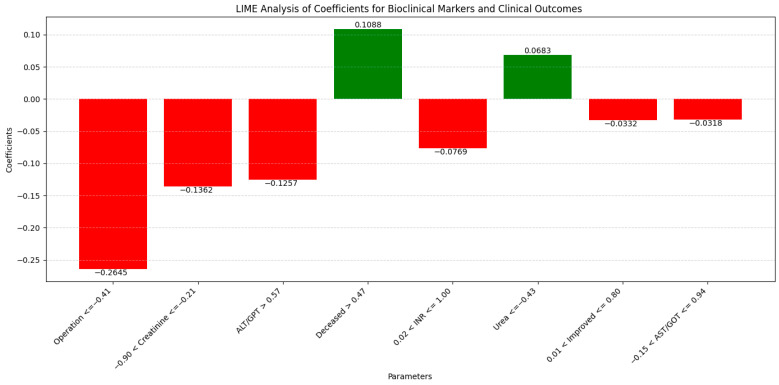
LIME analysis of coefficients of parameters affecting prediction: pre- and post-pandemic biochemical markers.

The LIME analysis results largely mirror those from the co-occurrence matrix, with both methods suggesting a stable influence of laboratory markers on clinical outcomes in the pre- and post-pandemic periods, with no significant differences between the periods. (Figure 16).

To streamline our analysis, we present the LIME values for the eight leading parameters.

The correlations of each parameter in LIME with the co-occurrence matrix are as follows:

Operation (−0.2645; ≤−0.41) indicated a moderate negative influence on mortality, consistent with observations from the co-occurrence matrix, where co-occurrence with recovery slightly decreased during the pandemic (0.38 pre-pandemic and 0.36 pandemic). This reflects that surgical interventions did not have a significantly different impact between periods.

Creatinine (−0.1362; −0.90 < Creatinine ≤ −0.21) showed a moderate negative influence as indicated by LIME, aligning with the observed decrease in co-occurrence with mortality in the matrix (0.32 pre-pandemic and 0.09 pandemic), suggesting a diminished impact of creatinine on mortality during the pandemic period.

Alanine Aminotransferase (ALT/GPT) (−0.1257; GPT > 0.57) demonstrated a moderate negative influence, in line with the co-occurrence matrix which indicates a slight increase in co-occurrence with mortality during the pandemic (0.22 pre-pandemic and 0.31 pandemic), suggesting a slight but insignificant change.

Deceased (0.1088; >0.47) indicated a positive influence on mortality, consistent with the increase observed in the matrix for several laboratory markers (e.g., INR and ALT), suggesting that mortality remained influenced by these factors without significant variations between periods.

International Normalized Ratio (INR) (−0.0769; 0.02 < INR ≤ 1.00) showed a slight negative influence as indicated by LIME, correlating with the insignificant increase trend of INR during the pandemic (co-occurrence with mortality increasing from 0.21 pre-pandemic to 0.31 pandemic), confirming a slight modification in impact.

Urea (0.0683; ≤−0.43) exhibited a small positive influence observed in LIME, consistent with the decrease observed in the co-occurrence matrix (0.34 pre-pandemic and 0.30 pandemic), highlighting that the impact of urea on mortality was relatively stable.

Improved (−0.0332; 0.01 < Improved ≤ 0.80) showed a slight negative influence, confirming the stability of improvements in the co-occurrence matrix, which did not show significant variations between periods for most markers.

Aspartate Aminotransferase (AST/GOT) (−0.0318; −0.15 < GOT ≤ 0.94) indicated a slight negative influence as shown by LIME, consistent with the increase in co-occurrence with mortality during the pandemic (0.15 pre-pandemic and 0.28 pandemic), suggesting that variations in AST did not have a significant impact.

Overall, these correlations highlight that the influences of laboratory markers on clinical outcomes remained stable and consistent between pre- and post-pandemic periods, without major significant differences, thereby validating the conclusions obtained from both the co-occurrence matrix and the LIME analysis.

### 3.6. Exploration of the Relationships between Specific Traumas and Clinical Outcomes

Our study explored the relationships between various types of abdominal trauma and the clinical outcomes of patients, including surgical interventions, reinterventions, recovery, improvement, and mortality. The analysis was conducted using co-occurrence matrices for the pre-pandemic and pandemic periods, providing insights into how these injuries influence the complexity of clinical cases.

**Figure 17:**
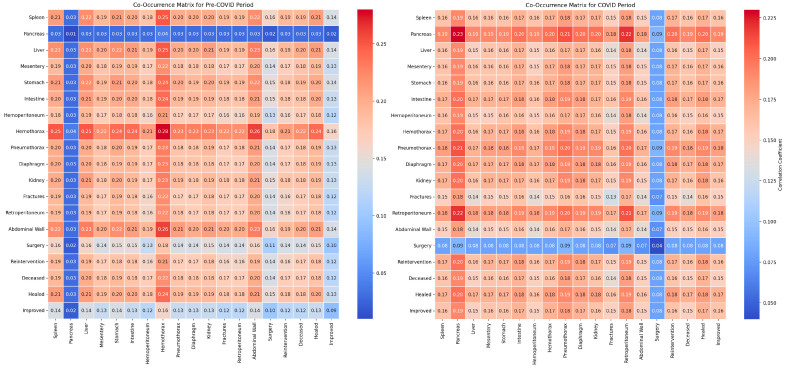
Comparative co-occurrence matrix of specific trauma correlations and management: pre-COVID versus COVID-19 pandemic periods.

The classical statistical analysis did not reveal statistically significant differences for splenic injuries between the periods (*p* = 0.210). The co-occurrence with the need for surgical intervention was 0.20 pre-pandemic and 0.18 during the pandemic, while the co-occurrence with mortality was 0.20 pre-pandemic and 0.18 during the pandemic (Figure 17).

For pancreatic injuries, the classical statistical analysis did not show significant differences between the periods, although the very small number of cases limited statistical significance. Co-occurrences increased from 0.03 to 0.17 for surgical intervention and from 0.03 to 0.18 for mortality between the pre-pandemic and pandemic periods.

The classical statistical analysis did not indicate statistically significant differences for hepatic injuries between the periods (*p* = 0.927), with co-occurrence with the need for surgical intervention being 0.21 pre-pandemic and 0.19 during the pandemic, and co-occurrence with mortality being 0.21 pre-pandemic and 0.19 during the pandemic.

Regarding mesenteric injuries, the classical statistical analysis did not show significant differences between the periods (*p* = 0.707). The co-occurrence with the need for surgical intervention was 0.20 pre-pandemic and 0.18 during the pandemic, while the co-occurrence with mortality was 0.20 pre-pandemic and 0.18 during the pandemic.

For gastric injuries, the classical statistical analysis did not reveal statistically significant variations between the periods (*p* = 0.957). The co-occurrence with the need for surgical intervention was 0.14 pre-pandemic and 0.07 during the pandemic, while the co-occurrence with mortality was 0.18 pre-pandemic and 0.15 during the pandemic.

The classical statistical analysis did not indicate statistically significant differences for intestinal injuries between the periods (*p* = 0.943), with co-occurrence with the need for surgical intervention being 0.19 pre-pandemic and 0.15 during the pandemic, and co-occurrence with mortality being 0.19 pre-pandemic and 0.16 during the pandemic.

For hemoperitoneum, the classical statistical analysis revealed a statistically significant difference between the periods (*p* = 0.016). The co-occurrence with the need for surgical intervention decreased from 0.19 pre-pandemic to 0.08 during the pandemic, while the co-occurrence with mortality decreased from 0.19 pre-pandemic to 0.17 during the pandemic.

The classical statistical analysis did not show statistically significant differences for hemothorax between the periods (*p* = 0.394). The co-occurrence with the need for surgical intervention was 0.16 pre-pandemic and 0.09 during the pandemic, while the co-occurrence with mortality was 0.20 pre-pandemic and 0.19 during the pandemic.

For pneumothorax, the classical statistical analysis did not show statistically significant variations between the periods (*p* = 0.784). The co-occurrence with the need for surgical intervention was 0.15 pre-pandemic and 0.08 during the pandemic, while the co-occurrence with mortality was 0.18 pre-pandemic and 0.17 during the pandemic.

The classical statistical analysis did not indicate statistically significant differences for diaphragmatic injuries between the periods (*p* = 0.706). The co-occurrence with the need for surgical intervention was 0.19 pre-pandemic and 0.08 during the pandemic, while the co-occurrence with mortality was 0.20 pre-pandemic and 0.17 during the pandemic.

For renal injuries, the classical statistical analysis did not show statistically significant differences between the periods (*p* = 0.635), with co-occurrence with the need for surgical intervention being 0.15 pre-pandemic and 0.08 during the pandemic, and co-occurrence with mortality being 0.19 pre-pandemic and 0.17 during the pandemic.

Regarding fractures, the classical statistical analysis did not indicate statistically significant differences between the periods (*p* = 0.740). The co-occurrence with the need for surgical intervention was 0.15 pre-pandemic and 0.08 during the pandemic, while the co-occurrence with mortality was 0.20 pre-pandemic and 0.17 during the pandemic.

The classical statistical analysis did not reveal statistically significant differences for retroperitoneal injuries between the periods (*p* = 0.921). The co-occurrence with the need for surgical intervention was 0.15 pre-pandemic and 0.08 during the pandemic, while the co-occurrence with mortality was 0.20 pre-pandemic and 0.17 during the pandemic.

For abdominal wall injuries, the classical statistical analysis did not show statistically significant differences between the periods (*p* = 0.844). The co-occurrence with the need for surgical intervention was 0.18 pre-pandemic and 0.08 during the pandemic, while the co-occurrence with mortality was 0.20 pre-pandemic and 0.19 during the pandemic.

**Figure 18:**
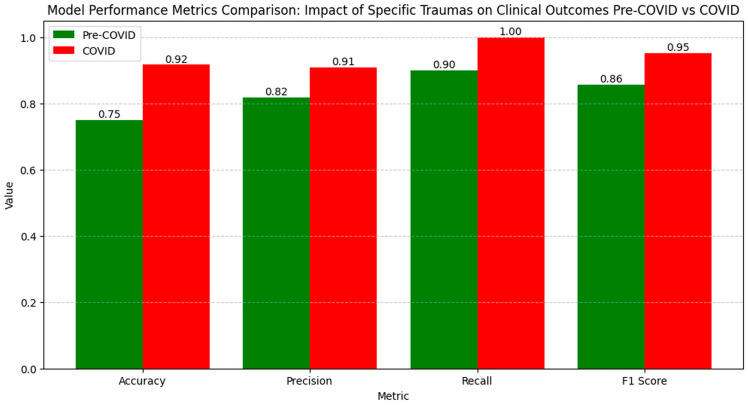
Model performance metrics on the impact of specific trauma on the management of abdominal trauma before and during the pandemic.

The numerical values of the metrics for evaluating the relationships between specific traumas and clinical outcomes show a clear improvement in model performance during the COVID period compared to the pre-COVID period (Figure 18).

Accuracy increased from 0.75 pre-COVID to 0.92 during the COVID period, suggesting an enhanced ability to correctly classify cases. Precision rose from 0.82 to 0.91, indicating a reduction in false positive results during the pandemic. Recall increased from 0.90 pre-COVID to 1.00 during the COVID period, demonstrating that the model identified all the relevant cases during the pandemic. The F1 Score increased from 0.86 pre-COVID to 0.95 during the COVID period, reflecting an improved balance between precision and recall, suggesting an optimized model performance in evaluating the influence of specific traumas on clinical outcomes during the pandemic period.

**Figure 19:**
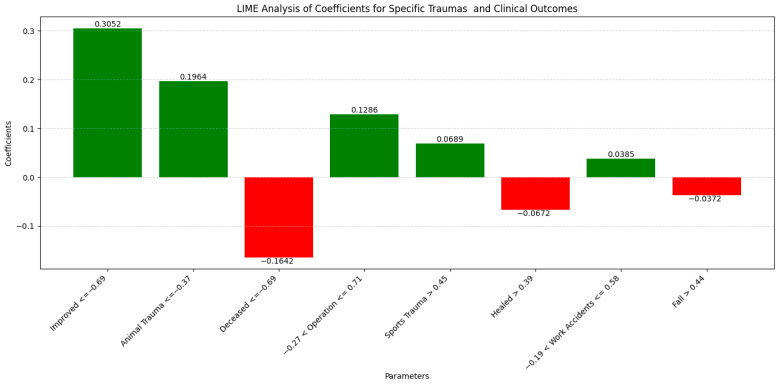
LIME analysis of coefficients of parameters affecting prediction: pre- and post-pandemic specific trauma.

The LIME analysis results largely mirror those from the co-occurrence matrix, with both methods suggesting a relatively stable influence of the different trauma types on clinical outcomes in the pre- and post-pandemic periods, with no major significant differences between the periods. In order to prevent information overload, we concentrate on the top eight parameters identified by LIME (Figure 19).

Specifically, Improved (0.3052; ≤−0.69) showed a moderate positive influence, aligning with the general stability observed in the co-occurrence matrix, where trauma-related co-occurrences did not show significant changes between the periods, suggesting a consistent impact of improvements on outcomes. Animal Trauma (0.1964; ≤−0.37) demonstrated a moderate positive influence, consistent with the matrix results that showed no significant differences for this trauma type, indicating that animal-origin traumas remained stable in their influence on surgical interventions and mortality. Deceased (−0.1642; ≤−0.69) indicated a negative influence on mortality, corresponding with the stability observed in mortality co-occurrences in the matrix for most traumas, confirming that mortality did not vary significantly between the periods. Operation (0.1286; −0.27 < Operation ≤ 0.71) exhibited a moderate positive influence in LIME, and aligned with the co-occurrence matrix results, where the necessity for surgical interventions remained constant or slightly decreased for several trauma types between the periods, reflecting a consistent impact. Sports Trauma (0.0689; >0.45) showed a slight positive influence, consistent with the matrix data indicating stable co-occurrences in sports-related traumas, even with a reduced number of cases during the pandemic. Healed (−0.0672; >0.39) demonstrated a negative influence, in line with the matrix observations where recovery co-occurrences remained stable for most trauma types, confirming that the healing process was not significantly influenced between the periods. Work Accidents (0.0385; −0.19 < Work Accidents ≤ 0.58) suggested a slight positive influence, consistent with the stability observed in the matrix for work-related traumas, where co-occurrences did not vary significantly between the periods. Fall (−0.0372; Fall > 0.44) indicated a negative influence, corresponding with the stability observed in the co-occurrence matrix for fall-related traumas, where no significant variations were observed in co-occurrences with mortality or the need for surgical interventions. Overall, these correlations confirm that the influences of the different trauma types on clinical outcomes remained relatively constant and consistent between the pre- and post-pandemic periods, validating the conclusions drawn from both the co-occurrence matrix and the LIME analysis.

## 4. Discussion

### 4.1. The Influence of Demographic Factors on Clinical Outcomes

Our study demonstrates that gender significantly affects clinical outcomes in abdominal trauma cases, aligning with the pre-pandemic findings by Peckham et al., who noted higher mortality and severe complications in males due to distinct immunological and physiological responses [21]. Utilizing a co-occurrence matrix analysis, we observed that during the COVID-19 pandemic, these gender disparities were reduced.

This shift may result from standardized treatment protocols implemented during the pandemic, as observed by Su et al., leading to uniform clinical interventions and reduced mortality differences between the genders [22]. Nguyen et al. suggest that the physiological adaptations induced by SARS-CoV-2 infection further leveled clinical responses, diminishing initial gender disparities [23].

Our co-occurrence analysis supports the hypothesis that the new physiological and immunological factors introduced by COVID-19 mitigated traditional gender-based differences in trauma response. Raza et al. support this notion, linking changes to standardized interventions and the virus’s complex effects on the immune system, particularly inflammatory responses [24]. Additionally, Biole et al. reported that males are more susceptible to severe COVID-19 complications, implying that pandemic treatment adaptations may have counteracted these vulnerabilities [25].

Ancochea et al. emphasize that early interventions and standardized protocols during the pandemic reduced gender disparities in post-trauma mortality, especially benefiting males [26]. Furthermore, Klein et al. and Raza et al. highlight that treatments during COVID-19 may have modulated hormonal differences impacting immunological responses, necessitating further research to understand these effects in abdominal trauma cases [24,27].

By incorporating co-occurrence matrix analysis, our findings underscore the pandemic’s complex impact on gender influences in clinical outcomes, offering new insights and directions for future research.

### 4.2. The Impact of Occupation and BMI on Abdominal Trauma in the Context of the COVID-19 Pandemic

Our study reveals significant lifestyle and work environment changes due to the COVID-19 pandemic, indirectly affecting obesity risk and the severity of abdominal trauma. Utilizing a co-occurrence analysis between occupation and BMI, we identified a strong association suggesting that occupation influences how obesity impacts both the severity and management of abdominal trauma. Alshahrani et al. confirmed that pandemic-induced lifestyle changes exacerbated the link between occupation and increased BMI [28].

The co-occurrence matrix showed this link was significant both before and during the pandemic, while the traditional statistical analysis did not reveal significant differences. This underscores the importance of co-occurrence analysis in uncovering hidden risk factors. Costello et al. supported this, indicating that occupational factors may impact obesity and trauma risk more than previously recognized, especially amid pandemic-induced work environment changes [29].

Our initial hypothesis was that the increased co-occurrence between occupation and BMI pre-pandemic, followed by a decrease during the pandemic, reflects forced adaptations in lifestyle and work conditions due to COVID-19. Bakaloudi et al. observed that remote work and altered physical demands significantly impacted BMI, leading to increased obesity, particularly due to reduced physical activity and dietary changes [30].

Regarding abdominal trauma severity, we suggest that obesity related to certain occupations influences not only trauma risk but also its complexity, especially when medical access is limited during crises. Nakeshbandi et al. highlighted obesity as a major risk factor for severe COVID-19 complications, exacerbating patient outcomes and increasing mortality, thereby amplifying vulnerability in abdominal trauma cases [31].

Safety measures during the pandemic may have mitigated some occupational risks while introducing new ones associated with sedentary behavior and lifestyle changes from remote work or altered job requirements. Katsoulis et al. discussed increased sedentary behavior’s negative health impact during this period, emphasizing vulnerability to severe complications, including abdominal trauma [32]. Hammouri et al. also noted that the pandemic led to significant BMI increases, heightening abdominal trauma risk [33].

Our study corroborates these findings, emphasizing the need for a deeper exploration of the links between occupation, obesity, and trauma risk during global crises. This could inform effective strategies for risk prevention and management in such contexts.

### 4.3. Age as a Predictive Factor for Clinical Outcomes in Abdominal Trauma During the COVID-19 Pandemic

Age significantly influences susceptibility to abdominal trauma and recovery capacity, especially amid the COVID-19 pandemic. Our co-occurrence analysis confirms that age remains an important predictor of mortality both before and during the pandemic, although the co-occurrence between age and mortality slightly decreased during this period.

Pre-pandemic studies by Imam et al. demonstrated that elderly patients with comorbidities such as cardiovascular diseases and diabetes have a significantly higher risk of mortality when infected with COVID-19 [34]. Mueller et al. also emphasized that individuals over 65 face a substantially higher risk of death compared to younger populations, underscoring age as a key mortality predictor [35].

We hypothesize that the slight decrease in co-occurrence between age and mortality during the pandemic may result from rapid adaptations in clinical protocols for elderly patients. Chinnadurai et al. noted that modifications in these protocols contributed to reduced mortality among elderly patients with high frailty [36]. De Smet et al. reported that while frailty and comorbidities remain significant predictive factors, adapted treatment strategies positively impacted clinical outcomes [37].

Prioritizing elderly patients in healthcare systems may have mitigated the impact of age on mortality. Koduri et al. discussed improvements emphasizing such prioritization, helping to reduce age-related mortality risks [38]. Kim et al. observed that older age groups benefited from early and prioritized interventions, reducing variations in mortality [39]. Additionally, Covino et al. suggested that comorbidities associated with COVID-19 may have altered the traditional relationships between age and clinical outcomes in abdominal trauma cases, contributing to the reduced influence of age on mortality during the pandemic [40].

Our study’s originality lies in using co-occurrence analysis to reveal nuanced shifts in age-related mortality patterns during the pandemic. These findings highlight the need for continued research to better understand the dynamics of age and comorbidities, aiming to develop clinical protocols that improve outcomes for elderly patients in both normal and crisis conditions.

### 4.4. The Impact of BMI on Treatment Duration and Outcomes During the COVID-19 Pandemic

Body mass index (BMI) is a critical factor in assessing risk and prognosis in abdominal trauma cases, especially during the COVID-19 pandemic. Our co-occurrence analysis revealed that BMI significantly influences hospital stay duration and mortality both before and during the pandemic, underscoring its role as a key predictor of trauma severity and clinical outcomes.

Suresh et al. demonstrated that patients with a BMI ≥ 30 kg/m^2^ more frequently require intensive care and mechanical ventilation, with longer hospital stays compared to those with a normal BMI [41]. Palaiodimos et al. identified obesity as a major risk factor for death in COVID-19 patients, with those having a BMI ≥ 35 kg/m^2^ experiencing nearly four times the mortality rate of individuals with normal BMI [42]. Similarly, Pranata et al. highlighted that each 5 kg/m^2^ increase in BMI significantly raises the risk of severe complications and mortality [43].

Obesity exacerbates patient outcomes and increases the need for intensive care resources. Du et al. found that a high BMI correlates with a greater likelihood of ICU admission and mechanical ventilation use, alongside a higher incidence of acute respiratory failure [44].

These findings emphasize the necessity for future research to focus on comparative longitudinal studies pre- and post-pandemic to explore obesity’s impact on clinical outcomes in abdominal trauma and COVID-19 cases. Bunnell et al. showed that visceral fat distribution influences disease severity, highlighting the importance of studying body composition changes [45]. Sjögren et al. suggested that systemic inflammation and visceral fat accumulation worsen the body’s response to severe infections like COVID-19, indicating a need for deeper investigation into these pathophysiological mechanisms [46].

To improve outcomes for obese patients, tailored clinical strategies are imperative. Klang et al. proposed that personalizing interventions, including adjusting medication dosages and intensive monitoring, can significantly enhance patient prognosis [47]. Developing specific clinical protocols for obese patients is essential for more effective management of these complex cases, reducing the burden on healthcare systems during the pandemic and beyond.

Our study’s originality lies in using co-occurrence analysis to highlight BMI as a major predictor of severity and clinical outcomes in abdominal trauma, emphasizing the continued importance of obesity as a critical factor in patient management.

### 4.5. Analysis of Biological and Laboratory Factors—Hemoglobin and Mortality

Our co-occurrence analysis identifies hemoglobin as a critical predictor of mortality in patients with abdominal trauma, with its influence notably increasing during the pandemic. This suggests that hemoglobin levels have become an even more vital indicator of death risk, possibly reflecting the metabolic and physiological deterioration induced by SARS-CoV-2 infection.

Tremblay et al. found anemia to be a significant predictor of mortality in COVID-19 patients, independent of age, sex, and comorbidities, reinforcing hemoglobin’s amplified role during this period [48]. Oh et al. demonstrated that patients with anemia upon admission had an increased mortality risk, indicating that early interventions based on hemoglobin monitoring, such as transfusions or oxygen therapy, could improve clinical outcomes—especially crucial when medical resources are limited [49].

Interpreting hemoglobin levels becomes more complex in patients with multiple comorbidities or those affected by COVID-19. Kuno et al. observed a U-shaped relationship between hemoglobin levels and mortality, where both low and high levels were associated with increased death risk [50]. This underscores the need to include hemoglobin monitoring as a central component of clinical management strategies, particularly for patients with abdominal trauma and complex comorbidities.

Future research should focus on hemoglobin’s impact on mortality in abdominal trauma cases. Mayneris-Perxachs et al. showed that hemoglobin can modulate the effects of hyperglycemia and obesity on death risk in COVID-19, highlighting the necessity for careful monitoring during the pandemic [51]. Additionally, Raman et al. proposed using the ferritin–hemoglobin ratio as a prognostic marker, offering an integrated approach to mortality risk assessment and clinical intervention optimization [52].

Our findings underscore hemoglobin’s heightened importance in prognosticating abdominal trauma outcomes during the COVID-19 pandemic. The careful monitoring and utilization of hemoglobin levels to guide clinical interventions could significantly improve patient outcomes and optimize resource allocation in global health crises.

### 4.6. Analysis of Biological and Laboratory Factors—Red Cell Distribution Width (RDW) and Mortality

Our co-occurrence analysis identified RDW as a significant marker correlated with mortality, with its co-occurrence increasing from the pre-pandemic to the pandemic period. This suggests that RDW has become an even more pertinent prognostic indicator in the context of COVID-19, reflecting systemic inflammation and metabolic dysfunction critical in severe trauma and viral infections like SARS-CoV-2.

The elevated co-occurrence between RDW and mortality likely indicates exacerbated oxidative stress and microvascular dysfunction amplified by the viral infection. Karampitsakos et al. confirmed that COVID-19 patients with RDW ≥ 14.5% had a significantly higher risk of mortality and disease progression, underscoring RDW’s amplified role during the pandemic [53].

RDW can serve as an early marker for assessing the severity of abdominal trauma in COVID-19 patients. Henry et al. demonstrated that RDW is a strong predictor of disease severity, capable of discriminating between different severity levels and predicting mortality [54]. These findings support using RDW to guide early clinical interventions, especially during crisis periods.

Variability in RDW may signal compromised erythrocyte homeostasis amid severe viral infections. Sarkar et al.’s meta-analysis showed that elevated RDW at admission is associated with increased mortality and severe disease progression in COVID-19 patients, highlighting its importance in risk stratification and personalized treatment [55].

To further validate RDW as a prognostic marker, future studies should explore its role in abdominal trauma during the pandemic. Wang et al. found RDW to be an independent predictor of COVID-19 severity, suggesting that integrating it into standard assessment and treatment protocols could improve patient prognosis [56].

Investigating the link between systemic inflammation, erythrocyte dysfunction (reflected by RDW), and clinical outcomes in abdominal trauma patients is crucial. Lucijanić et al. demonstrated that RDW is associated with increased mortality risk among both anemic and non-anemic COVID-19 patients, indicating its potential in risk stratification across various clinical contexts [57].

Longitudinal analyses of RDW variations and their correlation with mortality and morbidity are essential. Gowda et al. showed that RDW is a strong prognostic indicator of mortality in COVID-19 patients, emphasizing the need for early therapeutic interventions based on this marker [58].

Our co-occurrence analysis highlights RDW’s importance as a critical indicator of disease severity and mortality risk in both the pre-pandemic and pandemic periods. Integrating RDW into standard treatment protocols could optimize patient outcomes during crisis contexts, emphasizing the need for its careful monitoring in patient management.

### 4.7. Efficiency of the Emergency System During the COVID-19 Pandemic

Our co-occurrence analysis between surgical interventions and patient recovery indicates that the emergency system maintained, or even slightly improved, its efficiency during the COVID-19 pandemic. Despite a marginal decrease in co-occurrence from 0.38 pre-pandemic to 0.36 during the pandemic, this suggests effective adaptation to unprecedented pressures.

Rapid adjustments such as optimizing patient flow, enhancing triage protocols, and efficient resource allocation were crucial. Mozharovsky et al. reported that although postoperative mortality increased during the pandemic, strict triage and treatment protocols maintained surgical efficiency, with minimal mortality differences compared to pre-pandemic patients [59].

Digital technologies and telemedicine significantly contributed to these adaptations. Jacob et al. demonstrated that implementing an E-Handover system improved surgical efficiency, reduced waiting times, and upheld social distancing measures essential for infection control [60]. These innovations facilitated better coordination and resource management under crisis conditions.

The continuous training of medical staff and protocol updates enabled swift responses to new challenges. Rausei et al. observed that while emergency surgeries decreased by 41%, the quality of care was sustained through ongoing training and protocol adjustments [61].

Future research should evaluate emergency system efficiency during crises, focusing on specific adaptations for managing abdominal trauma patients. Osorio et al. found higher postoperative complications and failure-to-rescue rates in COVID-19 patients, underscoring the need for improved protocols [62]. Investigating the impact of technologies and telemedicine remains vital; Pecoraro et al. emphasized that efficient resource use was key during the pandemic, with regions possessing robust infrastructure managing emergencies more effectively [63].

Comparative studies on patient flow and resource allocation before and during the pandemic can identify best practices. Wong and Cheung noted a significant decrease in elective surgeries but maintained efficiency in emergencies, highlighting adaptability’s importance in resource management [64].

Our study’s originality lies in using co-occurrence analysis to reveal these insights, demonstrating that the emergency system effectively sustained surgical efficiency during the pandemic through rapid adaptations, digital technologies, and continuous staff training.

### 4.8. Future Perspectives and Clinical Implications of the Proposed Methodology

Integrating co-occurrence matrices with classical statistical analysis and machine learning offers an innovative approach to uncovering the complex relationships between demographic, biological, and clinical variables, advancing modern medical research. This methodology, especially during the COVID-19 pandemic, has proven superior in revealing subtle patterns and hidden associations that traditional methods might overlook.

By combining co-occurrence analysis with machine learning, we identified critical clinical dynamics not evident in conventional analyses. Shamout et al. demonstrated that machine learning models applied to electronic health records outperform traditional models in predicting clinical outcomes, highlighting the added value of these methods [65].

During the pandemic, our methodology facilitated the rapid and accurate analysis of complex clinical developments. Stevens et al. emphasize the importance of the transparent reporting of machine learning analyses in medicine to ensure correct understanding and critical evaluation by clinicians and researchers [66], which is essential for integrating new discoveries into practice.

A significant advantage of this approach is its potential to personalize medical care. MacKay et al. showed that machine learning-based risk models applied to health data improve the predictions of mortality and adverse events, enabling the nuanced evaluation of factors influencing clinical outcomes and paving the way for personalized treatments [67].

Future research should apply co-occurrence methodology in other fields like oncology or neurology to explore emerging clinical complexities. Lehmann et al. suggest that machine learning in oncology can provide new insights into patient outcomes and support personalized therapies [68].

Combining machine learning with co-occurrence analysis may discover new clinical relationships and prognostic indicators for treatment guidelines. Banerjee et al. found that these methods improve risk prediction and subtype definitions in cardiovascular diseases, emphasizing their importance for future research [69].

Risk stratification in populations with multiple comorbidities is another important direction. Ciarmiello et al. demonstrated that machine learning models using demographic and laboratory data can identify COVID-19 patients at risk of severe disease, highlighting the relevance of early, personalized interventions [70].

In conclusion, integrating co-occurrence analysis with machine learning represents a significant advancement in clinical research, offering unique perspectives to enhance medical practice. The originality of our method lies in detecting complex relationships and personalizing treatments, opening new avenues for effective, targeted interventions.

### 4.9. Addressing Gaps in Understanding Abdominal Trauma During the COVID-19 Pandemic

Despite extensive research on abdominal trauma and its management, the COVID-19 pandemic introduced unprecedented challenges that disrupted traditional care protocols and patient outcomes. A significant gap exists in the literature regarding the comprehensive analysis of how demographic, clinical, and laboratory factors interact to influence abdominal trauma outcomes during such global health crises. Previous studies have primarily focused on individual risk factors without exploring the complex interrelationships between them, especially in the pandemic context.

Our study fills this gap by employing an innovative methodology that integrates co-occurrence analysis with machine learning techniques. This approach allows for a more nuanced exploration of the interactions between variables such as age, gender, BMI, occupation, and hemoglobin levels, providing deeper insights into their collective impact on patient outcomes. By generating co-occurrence matrices through machine learning models, we identified subtle patterns and associations that traditional statistical methods might overlook.

For instance, we discovered that the co-occurrence between occupation and BMI revealed hidden risk factors related to lifestyle changes during the pandemic, which were not evident in classical analyses [28,29]. Similarly, the increased co-occurrence between hemoglobin levels and mortality highlighted the amplified role of hemoglobin as a prognostic marker in the pandemic context [48,49].

To ensure the robustness and generalizability of our findings, we implemented rigorous machine learning validation techniques, including an 80/20 train-test split and 5-fold cross-validation. This process minimized overfitting and enhanced the model’s ability to generalize relationships between the variables. We also utilized the SHAP (Shapley Additive Explanations) method to quantify the impact of each variable on clinical outcomes, providing the clear interpretability of our model’s predictions.

By addressing the literature gap on the complex interplay of the factors affecting abdominal trauma during the COVID-19 pandemic, our study offers valuable contributions to both research and clinical practice. The innovative use of co-occurrence analysis and machine learning provides a framework for future studies to explore multifaceted clinical scenarios more effectively. This methodology not only enhances our understanding of abdominal trauma in crisis contexts but also supports the development of targeted interventions to improve patient outcomes.

## 5. Study Limitations

This study has several limitations that should be considered when interpreting the results despite its significant contributions.

While the sample size of 118 patients is relatively small, it allowed for a detailed and specific examination of the data, facilitating the identification of relevant trends in the management of abdominal trauma in a pandemic context. Although this sample size may limit the generalizability of the results to larger populations and may affect the stability of statistical analyses, the study provides a valuable foundation for exploratory and pilot research in this field.

The time period analyzed, covering both the pre-pandemic stage and the COVID-19 pandemic period, provides an important perspective on the direct impact of the global health crisis on medical management. Although the subsequent events are not included, the chosen timeframe is essential for understanding the immediate effects of the pandemic.

Selection bias, while it may limit the applicability of the results to more diverse populations, contributes to the coherence and homogeneity of the data, enhancing the relevance of the findings for the studied group.

The exclusion of patients with incomplete data and the presence of confounding variables are common limitations in medical research, underscoring the importance of rigorous data collection and analysis methods, as well as the need for the careful control of these factors in future studies.

External factors may complicate the interpretation of results but also provide a realistic context for evaluating the management of abdominal trauma under crisis conditions, reflecting the complex realities faced by healthcare professionals.

The use of machine learning techniques, although it carries risks of overfitting and interpretative challenges, has brought new perspectives to data analysis, contributing to advancing the understanding of this field.

In conclusion, while the study presents certain limitations, its findings remain valuable and relevant, underscoring the need to continue research in this area to expand and validate the conclusions presented, thereby contributing to the optimization of medical practices in the face of global challenges.

## 6. Conclusions

Our study offers an innovative perspective on abdominal trauma management during the COVID-19 pandemic by utilizing co-occurrence analysis enhanced with machine learning techniques. This approach allowed us to uncover the complex and previously hidden relationships between clinical and demographic variables that traditional methods might overlook.

We found that factors such as age, sex, BMI, occupation, and RDW significantly influenced patient outcomes. The co-occurrence analysis revealed that the standardization of clinical protocols and physiological adaptations associated with SARS-CoV-2 contributed to reducing mortality differences between the sexes. Additionally, the link between occupation and obesity underscored the influence of the work environment on trauma severity, particularly during the pandemic when lifestyle changes impacted BMI and, consequently, patient outcomes. RDW emerged as an important prognostic marker, reflecting disease severity and the increased risk of mortality in the context of COVID-19, highlighting the need for careful monitoring in patient management.

Despite the pressures imposed by the pandemic, our findings suggest that the emergency healthcare system adapted effectively, maintaining or even improving the efficiency of surgical interventions. The stable co-occurrence between surgical interventions and patient recovery indicates that rapid adaptations, the use of digital technologies, and the continuous training of medical staff were essential in sustaining high standards of care.

By employing advanced analytical methodologies, our study successfully uncovered subtle patterns and interactions among variables, offering valuable insights for optimizing medical management during global crises. These findings enhance our understanding of how the pandemic influenced clinical approaches and emphasize the importance of developing flexible and effective strategies to improve patient outcomes in similar contexts.

Future research should delve deeper into the role of prognostic markers like RDW and continue developing personalized clinical strategies that enhance patient care during global crises. By focusing on the complex interplay of demographic and clinical factors revealed through co-occurrence analysis, healthcare systems can strengthen their capacity to respond effectively under extreme stress conditions, ultimately improving patient outcomes.

## Figures and Tables

**Table 1:** Comparative analysis of abdominal trauma variables pre-COVID vs. COVID period.

Variable Name	Variable Type	Number and Percentage Pre-COVID Period	Number and Percentage COVID Period	Chi-Square Value	*p*-Value
Sex	M	47 (53.41%)	41 (46.59%)	0.5504	0.458
	F	13 (43.33%)	17 (56.67%)		
Occupation	Yes	9 (45.00%)	11 (55.00%)	0.108	0.742
	No	51 (52.04%)	47 (47.96%)		
Penetrating	Yes	13 (52.00%)	12 (48.00%)	0.0091	0.923
	No	47 (50.54%)	46 (49.46%)		
Self-injury	Yes	6 (46.15%)	7 (53.85%)	0.0042	0.948
	No	54 (51.43%)	51 (48.57%)		
Work accident	Yes	1 (50.00%)	1 (50.00%)	0.0006	0.980
	No	59 (50.86%)	57 (49.14%)		
Violence	Yes	10 (43.48%)	13 (56.52%)	0.3085	0.578
	No	50 (52.63%)	45 (47.37%)		
Animal-related	Yes	4 (50.00%)	4 (50.00%)	0.1002	0.751
	No	56 (50.91%)	54 (49.09%)		
Sports-related	Yes	1 (100.00%)	0	-	-
	No	59 (50.43%)	58 (49.57%)		
Fall	Yes	18 (54.55%)	15 (45.45%)	0.0873	0.767
	No	42 (49.41%)	43 (50.59%)		
Road traffic	Yes	19 (51.35%)	18 (48.65%)	0.0155	0.900
	No	41 (50.62%)	40 (49.38%)		
Hepatitis	Yes	6 (75.00%)	2 (25.00%)	1.1005	0.294
	No	54 (49.09%)	56 (50.91%)		
Diabetes	Yes	3 (33.33%)	6 (66.67%)	0.5575	0.455
	No	57 (52.29%)	52 (47.71%)		
Obesity	Yes	8 (33.33%)	16 (66.67%)	2.8703	0.090
	No	52 (55.32%)	42 (44.68%)		
Fibrillation	Yes	3 (37.50%)	5 (62.50%)	0.173	0.677
	No	57 (51.82%)	53 (48.18%)		
Hypertension	Yes	7 (31.82%)	15 (68.18%)	3.038	0.081
	No	53 (55.21%)	43 (44.79%)		
Cogestive hearth failure	Yes	4 (50.00%)	4 (50.00%)	0.1002	0.751
	No	56 (50.91%)	54 (49.09%)		
Kidney Failure	Yes	3 (50.00%)	3 (50.00%)	0.1417	0.706
	No	57 (50.89%)	55 (49.11%)		
Alchoholism	Yes	4 (40.00%)	6 (60.00%)	0.1495	0.699
	No	56 (51.85%)	52 (48.15%)		
Surgery	Yes	51 (48.11%)	55 (51.89%)	2.135	0.144
	No	9 (75.00%)	3 (25.00%)		
Spleen	Yes	23 (60.53%)	15 (39.47%)	1.569	0.210
	No	37 (46.25%)	43 (53.75%)		
Pancreas	Yes	0 (0%)	2 (100.00%)	-	-
	No	60 (51.72%)	56 (48.28%)		
Liver	Yes	11 (47.82%)	12 (52.17%)	0.0082	0.927
	No	49 (51.58%)	46 (48.42%)		
Mesentery	Yes	4 (66.67%)	2 (33.33%)	0.1417	0.707
	No	56 (50.00%)	56 (50.00%)		
Stomach	Yes	5 (55.56%)	4 (44.44%)	0.0028	0.957
	No	55 (50.46%)	54 (49.54%)		
Intestine	Yes	8 (53.33%)	7 (46.67%)	0.0049	0.943
	No	52 (50.49%)	51 (49.51%)		
Hemoperitoneum	Yes	35 (63.64%)	20 (36.36%)	5.817	0.016 *
	No	25 (39.68%)	38 (60.32%)		
Hemothorax	Yes	8 (66.67%)	4 (33.33%)	0.726	0.394
	No	52 (49.06%)	54 (50.94%)		
Pneumothorax	Yes	6 (60.00%)	4 (40.00%)	0.0754	0.784
	No	54 (50.00%)	54 (50.00%)		
Diaphragm	Yes	3 (50.00%)	3 (50.00%)	0.1417	0.706
	No	57 (50.89%)	55 (49.11%)		
Kidney	Yes	3 (75.00%)	1 (25.00%)	0.2249	0.635
	No	57 (50.00%)	57 (50.00%)		
Fractures	Yes	21 (47.73%)	23 (52.27%)	0.1105	0.740
	No	39 (52.70%)	35 (47.30%)		
Retroperitoneum	Yes	9 (52.94%)	8 (47.06%)	0.009	0.921
	No	51 (50.50%)	50 (49.50%)		
Abdominal Wall	Yes	7 (53.85%)	6 (46.15%)	0.0385	0.844
	No	53 (50.96%)	51 (49.04%)		
Awarenes	Yes	44 (48.35%)	47 (51.65%)	0.6028	0.438
	No	16 (59.26%)	11 (40.74%)		
Pain	Yes	43 (48.31%)	46 (51.69%)	0.5629	0.453
	No	17 (58.62%)	12 (41.38%)		
Reintervention	Yes	1 (20.00%)	4 (80.00%)	0.908	0.341
	No	59 (52.21%)	54 (47.79%)		
Condition	Deceased	11 (55.00%)	9 (45.00%)	6.450	0.039 *
	Recovered	44 (56.41%)	34 (43.58%)		
	Improved	5 (25.00%)	25 (75.00%)		

* *p* < 0.05—statistically significant.

**Table 2:** Results of the univariate and multivariate logistic regression analysis for the pre- and post-pandemic periods.

Variable	Univariate Analysis OR (95% CI)	*p*-Value	Multivariate Analysis OR (95% CI)	*p*-Value
Sex	1.50 (0.65, 3.45)	0.3419		
Occupation	0.75 (0.29, 1.98)	0.5667		
Penetrating	1.06 (0.44, 2.57)	0.8967		
Self-injury	0.81 (0.25, 2.57)	0.7201		
Work accident	0.97 (0.06, 15.82)	0.9807		
Violence	0.69 (0.28, 1.73)	0.4321		
Animal-related	0.96 (0.23, 4.05)	0.9604		
Sports-related	3.50 × 10^9^ (0.00, Inf)	0.9997		
Fall	1.23 (0.55, 2.75)	0.6169		
Road traffic	1.03 (0.47, 2.24)	0.9410		
Hepatitis	3.11 (0.60, 16.09)	0.1759		
Diabetes	0.46 (0.11, 1.92)	0.2840		
Obesity	0.40 (0.16, 1.03)	0.0590		
Fibrillation	0.56 (0.13, 2.45)	0.4394		
Hypertension	0.38 (0.14, 1.01)	0.0529		
Congestive heart failure	0.96 (0.23, 4.05)	0.9660		
Kidney Failure	0.63 (0.10, 3.93)	0.6227		
Alcoholism	0.96 (0.19, 4.99)	0.9660		
Surgery	0.31 (0.08, 1.21)	0.0909		
Spleen	1.78 (0.81, 3.91)	0.1492		
Pancreas	0.00 (0.00, Inf)	0.9889		
Liver	0.96 (0.38, 2.42)	0.9298		
Mesentery	2.00 (0.35, 11.36)	0.4342		
Stomach	1.23 (0.31, 4.82)	0.7691		
Intestine	1.12 (0.38, 3.32)	0.8367		
Hemoperitoneum	2.66 (1.26, 5.61)	0.0102 *	2.82 (1.29, 6.18)	0.0093 *
Hemothorax	2.08 (0.59, 7.32)	0.2553		
Pneumothorax	1.50 (0.40, 5.62)	0.5472		
Diaphragm	0.96 (0.19, 4.99)	0.9660		
Kidney	3.00 (0.30, 29.71)	0.3477		
Fractures	0.82 (0.39, 1.73)	0.6013		
Retroperitoneum	1.10 (0.39, 3.09)	0.8520		
Abdominal Wall	1.14 (0.36, 3.64)	0.8188		
Awareness	0.64 (0.27, 1.54)	0.3213		
Pain	0.66 (0.28, 1.54)	0.3365		
Reintervention	0.23 (0.02, 2.11)	0.1933		
Condition	0.82 (0.51, 1.31)	0.4011		

* *p* < 0.05—statistically significant. The multivariate analysis included the variables hemoperitoneum, obesity, hypertension, surgery, and sex.

**Table 3:** Comparative analysis of medical and biological data between pre-COVID and COVID periods.

Variable Name	Median (IQR) Pre-COVID	Median (IQR) COVID Period	*p* Value
Age	38.0 (27.0, 63.0)	45.0 (37.5, 60.8)	0.110
BMI	85.1 (80.0, 85.1)	85.1 (72.2, 85.1)	0.292
Blood	1.3 (0.3, 2.2)	0.9 (0.4, 1.9)	0.550
Hosp. Days	7.0 (3.0, 10.0)	6.0 (3.0, 8.0)	0.188
Hours	14.4 (9.9, 18.5)	13.5 (9.5, 16.5)	0.267
Time	3.0 (1.1, 12.2)	2.4 (1.3, 6.4)	0.789
Number	10.0 (9.0, 11.0)	11.0 (10.0, 11.0)	0.029 *
Hb	11.9 (9.6, 13.3)	11.7 (10.0, 13.5)	0.948
Hb/Hct	3.0 (2.9, 3.1)	3.0 (2.9, 3.1)	0.291
Hct	34.7 (27.9, 39.5)	34.9 (30.4, 39.9)	0.576
Urea	33.5 (26.0, 39.8)	34.0 (25.2, 39.8)	0.850
Creatinine	0.8 (0.7, 1.0)	0.8 (0.6, 1.1)	0.712
INR	1.1 (1.1, 1.3)	1.1 (1.0, 1.2)	0.067
GPT	42.5 (26.5, 133.0)	43.5 (22.0, 163.3)	0.661
GOT	51.0 (30.0, 121.8)	68.5 (30.5, 157.0)	0.486
Neutrophils	80.3 (72.9, 85.3)	80.4 (73.4, 86.0)	0.739
Monocytes	7.7 (5.6, 8.4)	7.5 (5.3, 8.5)	0.775
Lymphocytes	11.8 (6.8, 16.8)	10.5 (6.2, 16.1)	0.557
Platelets	176.5 (125.0, 225.4)	190.0 (156.3, 245.5)	0.265
Leukocytes	12.4 (9.1, 16.0)	14.1 (11.1, 17.1)	0.149
Erythrocytes	3.8 (3.1, 4.2)	3.9 (3.1, 4.4)	0.421
MCV	89.0 (86.5, 92.7)	90.7 (87.5, 95.7)	0.103
RDW	12.5 (11.9, 13.4)	13.2 (12.4, 14.0)	0.006 *
Sodium	139.0 (136.8, 141.0)	139.0 (136.0, 141.0)	0.545
Potassium	4.1 (3.9, 4.4)	4.2 (3.8, 4.4)	0.976
Glucose	118.5 (94.8, 169.3)	121.0 (99.2, 154.0)	0.626
Amylase	55.0 (45.0, 87.3)	54.0 (39.0, 78.5)	0.353
NLR	6.5 (4.4, 12.4)	7.6 (4.4, 13.6)	0.732
IIC	7.0 (4.8, 15.3)	8.8 (6.0, 16.2)	0.339
MCVL	7.6 (5.4, 13.3)	8.2 (5.7, 13.3)	0.559

* *p* < 0.05—statistically significant; Mann–Whitney U test.

## Data Availability

Data is contained within the article: The original contributions presented in the study are included in the article, further inquiries can be directed to the corresponding authors.
